# Multiplexed Metagenomic Deep Sequencing To Analyze the Composition of High-Priority Pathogen Reagents

**DOI:** 10.1128/mSystems.00058-16

**Published:** 2016-07-19

**Authors:** Michael R. Wilson, Greg Fedewa, Mark D. Stenglein, Judith Olejnik, Linda J. Rennick, Sham Nambulli, Friederike Feldmann, W. Paul Duprex, John H. Connor, Elke Mühlberger, Joseph L. DeRisi

**Affiliations:** a Department of Neurology, University of California, San Francisco, San Francisco, California, USA; b Department of Biochemistry and Biophysics, University of California, San Francisco, San Francisco, California, USA; c Integrative Program in Quantitative Biology, Bioinformatics, University of California, San Francisco, San Francisco, California, USA; d Department of Microbiology, Immunology, and Pathology, Colorado State University, Fort Collins, Colorado, USA; e Department of Microbiology, Boston University School of Medicine, Boston, Massachusetts, USA; f National Emerging Infectious Diseases Laboratories, Boston University, Boston, Massachusetts, USA; g Rocky Mountain Veterinary Branch, National Institute of Allergy and Infectious Diseases, National Institutes of Health, Hamilton, Montana, USA; h Howard Hughes Medical Institute, Chevy Chase, Maryland, USA; Mayo Clinic

**Keywords:** metagenomics, pathogen tracking, phylogenetic analysis

## Abstract

Laboratories studying high-priority pathogens need comprehensive methods to confirm microbial species and strains while also detecting contamination. Metagenomic deep sequencing (MDS) inventories nucleic acids present in laboratory stocks, providing an unbiased assessment of pathogen identity, the extent of genomic variation, and the presence of contaminants. Double-stranded cDNA MDS libraries were constructed from RNA extracted from *in vitro*-passaged stocks of six viruses (La Crosse virus, Ebola virus, canine distemper virus, measles virus, human respiratory syncytial virus, and vesicular stomatitis virus). Each library was dual indexed and pooled for sequencing. A custom bioinformatics pipeline determined the organisms present in each sample in a blinded fashion. Single nucleotide variant (SNV) analysis identified viral isolates. We confirmed that (i) each sample contained the expected microbe, (ii) dual indexing of the samples minimized false assignments of individual sequences, (iii) multiple viral and bacterial contaminants were present, and (iv) SNV analysis of the viral genomes allowed precise identification of the viral isolates. MDS can be multiplexed to allow simultaneous and unbiased interrogation of mixed microbial cultures and (i) confirm pathogen identity, (ii) characterize the extent of genomic variation, (iii) confirm the cell line used for virus propagation, and (iv) assess for contaminating microbes. These assessments ensure the true composition of these high-priority reagents and generate a comprehensive database of microbial genomes studied in each facility. MDS can serve as an integral part of a pathogen-tracking program which in turn will enhance sample security and increase experimental rigor and precision.

**IMPORTANCE** Both the integrity and reproducibility of experiments using select agents depend in large part on unbiased validation to ensure the correct identity and purity of the species in question. Metagenomic deep sequencing (MDS) provides the required level of validation by allowing for an unbiased and comprehensive assessment of all the microbes in a laboratory stock.

## Abstract (precis)

Both the integrity and reproducibility of experiments using select agents depend in large part on unbiased validation to ensure the correct identity and purity of the species in question. Metagenomic deep sequencing (MDS) provides the required level of validation by allowing for an unbiased and comprehensive assessment of all the microbes in a laboratory stock.

## INTRODUCTION

Virology laboratories must maintain constant vigilance regarding the provenance of strains in their collections. In addition to confirming that experiments are being performed on the intended virus, unbiased methods that can detect viral coinfections and/or other microbial contaminants are critical for ensuring that experiments are faithfully reporting on the biology of a particular virus and not on a polymicrobial exposure. Stock assurance is exceptionally important when working with viruses which require a high level of biocontainment, given the increased security requirements and higher costs and higher stakes of the research.

The validity and reproducibility of experiments, in addition to security and tracking concerns, demand an approach that can provide not only simple viral identification but also the entire spectrum of single nucleotide variants (SNVs) and their respective frequencies within a given stock. Molecular subtyping was critical in determining the source of the 2001 bioterrorism-associated anthrax outbreak (1). Furthermore, the recent missteps in the handling of *Bacillus anthracis*, influenza virus, and smallpox virus highlight the potential for inadvertent laboratory contamination and the need to improve isolate-tracking methods (2, 3).

In this study, we sequenced virus stocks of six distinct negative-sense RNA viruses, each of them with a unique passage history: (i) mycoplasma-contaminated Ebola virus (EBOV), (ii) mycoplasma-contaminated La Crosse virus (LACV) grown on cells with a known lymphocytic choriomeningitis virus (LCMV) contamination, (iii) vesicular stomatitis virus (VSV) grown on LCMV-contaminated cells, (iv) recombinant human respiratory syncytial virus (rHRSV) expressing enhanced green fluorescent protein (EGFP) from an additional transcription unit (ATU) (4), (v) recombinant canine distemper virus (rCDV) expressing Venus fluorescent protein from an ATU (5), and recombinant measles virus (rMV) expressing EGFP from an ATU (6). The investigators performing the sequence analysis were blinded to the virus isolates and the origins and known contaminations of the virus stocks. Here, we demonstrate the utility of metagenomic deep sequencing (MDS) for comprehensively assessing multiple viral isolates. We show that MDS and custom bioinformatics pipelines utilizing publicly available and free software packages can detect a wide range of bacterial and viral contaminants in high-priority virus stocks and that SNV analysis can successfully discriminate between two closely related EBOV isolates. We also demonstrate how dual-indexed barcodes dramatically decrease the false-positive assignment of sequencing reads to an input sample in multiplexed high-titer virus MDS libraries.

## RESULTS

### Importance of dual-index barcoding.

In many sequencing applications, including MDS, it is critical to be able to distinguish low-level true-positive samples from false positives that result from assignment of sequencing reads to the wrong index (“index bleed-through”). Chimeric molecules produced during amplification steps in library preparation or on the sequencer during cluster generation are particularly susceptible to such misassignment. Creating library molecules with barcode sequences on both ends of the molecule (dual indexing) has been shown to reduce the rate at which this misassignment occurs by requiring concordance between the two barcode sequences (7).

**FIG 1:**
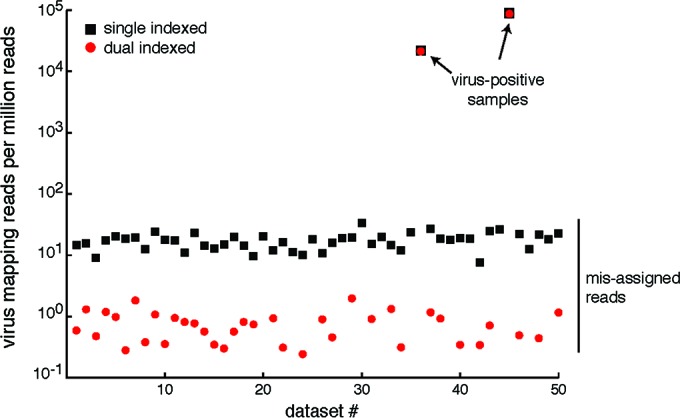
Dual indexing decreases the median rate of read misassignment by nearly 40-fold. Libraries from 50 samples were pooled and sequenced together. Two of the samples (indicated by arrows) were positive for snake arenavirus, and the other 48 were negative. Data sets were demultiplexed using a single index sequence (black squares) or dual index sequences (red circles), and reads from each data set were mapped with high stringency to the virus sequence. The number of virus mapping reads per million quality-filtered reads is indicated. Some dual-index-demultiplexed data sets had no misassigned reads. In these cases, red circles are not shown.

To corroborate the benefit of minimizing misassignment of sequences by using dual indexing in the context of MDS, we analyzed the rate of index bleed-through in an independently generated sequencing data set that was demultiplexed using one or both index reads. We measured the rate of bleed-through in a set of 50 samples that included two samples that were positive for a recently discovered reptarenavirus. The other 48 samples were negative by quantitative reverse transcription PCR (qRT-PCR). We aligned reads from all 50 pooled samples to a virus genome segment present in the two positive samples (8). The data sets contained a median of 2.75 × 10^6^ read pairs, and the two positive samples contained 65,284 and 203,565 virus-mapping reads (Fig. 1). Index bleed-through was determined to have occurred when reads identified as one of the 48 virus-negative samples by barcode actually aligned to this particular reptarenavirus genome segment. The median rate of bleed-through in the data sets that were demultiplexed using a single index was 17.9 per million filtered reads (Fig. 1). The median rate in dual-index demultiplexed data sets was 0.48 per million reads. Thus, in our data set, dual indexing reduced the median rate of read misassignment by 37-fold. The absolute number of mismapping reads decreased from a median of 50 reads to one read per data set.

### Identification of microbes.

The MDS of total RNA from each sample yielded 150-nucleotide (nt) paired-end sequences (Table 1). The data were analyzed using a rapid computational pipeline developed at the University of California, San Francisco (UCSF), to classify MDS reads and identify potential pathogens by comparison to the entire NCBI nucleotide reference database as described in Materials and Methods (9).

**TABLE 1:** Metagenomic deep sequencing results^a^

Sample	Total no. of sequencing reads	No. of unique nonhuman reads	Target virus	Other viral sequence(s)	Bacterial sequence
EBOV	47,328,387	17,777,406	EBOV (3,342,624)	None	*Mycoplasma hyorhinis* (12,215,655)
rMV	7,129,497	802,549	MV (159,184)	HHV4 (905)	Negative
rHRSV	6,274,384	1,165,688	HRSV (673,096)	HPV18 (121)	Negative
LACV	6,217,368	1,389,113	LACV (1,122,903)	LCMV (5,388), Syrian hamster IAP H10 (45), hamster gammaretrovirus (11)	*Mycoplasma arginini* (23,919)
rCDV	6,165,559	900,609	CDV (582,074)	Fowlpox (1,138), LACV (7)	Negative
VSV	10,164,946	2,130,521	VSV (1,481,456)	LCMV (3,315)	Negative

a Number of sequences aligning to each microbe are in parentheses. Abbreviations: EBOV, Ebola virus; rMV, recombinant measles virus; HHV4, human herpesvirus 4; rHRSV, recombinant human respiratory syncytial virus; LACV, La Crosse virus; HPV18, human papillomavirus 18; IAP, intracisternal A particle; LCMV, lymphocytic choriomeningitis virus; rCDV, recombinant canine distemper virus; VSV, vesicular stomatitis virus.

The results are summarized in Table 1. The EBOV and LACV samples were found to have significant contamination with *Mycoplasma* spp., with 3.65 and 0.02 *Mycoplasma* reads per viral read, respectively. The rMV sample contained more than 900 nonredundant, paired-end Epstein-Barr virus (EBV) sequences (0.013% of total reads) that were expected to come from the EBV-transformed human B-lymphoblastoid cell line (B-LCL) on which the virus was grown (10). Similarly, the rHRSV sample contained 121 paired-end sequences (0.0019% of total reads) that aligned to human papillomavirus 18 (HPV18). This was also unsurprising, since HEp-2 cells are the standard cell line used to culture HRSV and these are known to be a HeLa‑contaminated cell line; HeLa cells were recently shown to have HPV18 DNA integrated into their genome (11). The LACV sample had evidence of Syrian hamster retroviruses, and it was confirmed that the virus had been grown on baby hamster kidney (BHK) cells. The rCDV sample contained sequence reads mapping to fowlpox virus, which had been used as part of the process to generate rCDV from plasmid. Because these RNA extractions were not DNase treated, we cannot rule out that some of the EBV, HPV18, and fowlpox virus sequences were remnants of genomic DNA. Last, the LACV and VSV samples were found to be contaminated with LCMV. None of the isolates had evidence of fungal contamination.

As evidence of the minimal index bleed-through in these dual-indexed samples, only seven viral read pairs unique to LACV were found in the rCDV sample, and no other sample cross-contamination was present in any of the other samples in this set, despite being processed in parallel. Regardless, the fact that low-level index bleed-through may still occur, despite dual indexing, highlights the need for additional layers of error correction. Future iterations of this technology will likely include added features that will further reduce bleed-through of multiplexed samples, such as unique molecular identifier (UMI) barcodes, in which each cDNA molecule is uniquely indexed at the time of first-strand synthesis (12).

### SNV experiment.

**FIG 2:**
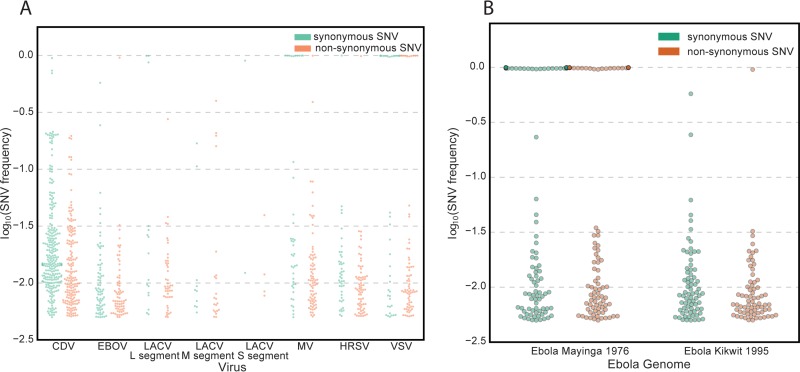
Distribution of single nucleotide variants (SNVs) for each virus and for two Ebola virus strains. For each plot, the *y* axis is the log_10_(SNV frequency) in order to display the range of low-frequency SNVs more accurately. (A) SNV analysis for each virus sample using its reference genome. Abbreviations: CDV, canine distemper virus; EBOV, Ebola virus; LACV, La Crosse virus; MV, measles virus; HRSV, human respiratory syncytial virus; VSV, vesicular stomatitis virus. (B) SNV analysis of 2,501,8050 filtered read-pairs revealed 223 SNVs in the reads that mapped to Ebola virus Mayinga with a frequency of ≥0.90, while there was only one SNV with a frequency of ≥0.90 in the reads that mapped to Ebola virus Kikwit.

All of the viruses contained several SNVs compared to their reference sequences that ranged in frequency, including some consensus-level SNVs, as listed in Table 2. At a frequency of greater than 0.005, rCDV had 427 SNVs (170 of those being nonsynonymous), EBOV had 143 SNVs (70 nonsynonymous), the LACV L segment had 61 SNVs (45 nonsynonymous), the LACV M segment had 31 SNVs (21 nonsynonymous), the LACV S segment had 6 SNVs (4 nonsynonymous), rMV had 122 SNVs (80 nonsynonymous), rHRSV had 115 SNVs (71 nonsynonymous), and VSV strain Indiana (VSV^IN^) had 109 SNVs (72 nonsynonymous). A majority of SNVs are rare variants, having a frequency of less than 0.10. All six viruses had nonsynonymous and synonymous SNVs at frequencies of greater than 0.10. Four viruses had nonsynonymous SNVs at a frequency of at least 0.90, while five viruses had synonymous SNVs at a frequency of at least 0.90. Figure 2A displays the distribution of the SNV frequency on each genomic segment.

**TABLE 2:** SNVs in each virus genome segment that are at least 0.5% of population^a^

Viral genome	Mean coverage	No. of SNVs:				
		Total	≥1%	≥10%	≥50%	≥90%
EBOV	8,334	143 (70)	49 (19)	3 (1)	2 (1)	1 (1)
MV	1,622	122 (80)	72 (42)	13 (2)	11 (1)	11 (1)
HRSV	5,429	115 (71)	54 (27)	2 (1)	2 (1)	2 (1)
LACV L segment	5,539	61 (45)	27 (18)	4 (1)	3 (0)	2 (0)
LACV M segment	10,077	31 (21)	11 (8)	6 (4)	0 (0)	0 (0)
LACV S segment	14,906	6 (4)	4 (2)	1 (0)	1 (0)	1 (0)
CDV	4,741	427 (170)	297 (91)	30 (6)	3 (0)	1 (0)
VSV	13,401	109 (72)	53 (31)	24 (10)	24 (10)	24 (10)

a Mean coverage is the mean number of non-PCR-duplicate reads that mapped to each base of the virus genome segment. Each SNV column is listed as the number of total SNVs, followed by the number of nonsynonymous SNVs in parentheses, which are at least the percentage of the population listed in the header. Abbreviations: EBOV, Ebola virus; MV, measles virus; HRSV, human respiratory syncytial virus; LACV, La Crosse virus; CDV, canine distemper virus; VSV, vesicular stomatitis virus.

The investigators were blinded to the exact isolate of EBOV since the major goal of this study was to identify a state-of-the-art approach which could precisely characterize a virus reagent with isolate specificity in an unbiased fashion. As described in Materials and Methods, SNV analysis was performed to determine the EBOV isolate. Reads were mapped to two different reference genomes: (i) Zaire ebolavirus isolate Ebola virus/*Homo sapiens*-tc/COD/1976/Yambuku-Mayinga, complete genome strain (GenBank accession no. NC_002549), and (ii) Zaire ebolavirus strain Kikwit, complete genome (GenBank accession no. JQ352763). Comparing rates of correctly mapped reads did not reveal isolate identity. The same 2,501,8050 filtered read-pairs mapped at virtually identical percentages, 21.03% to EBOV Mayinga and 21.05% to EBOV Kikwit. However, SNV analysis revealed 223 SNVs in the reads that mapped to EBOV Mayinga with a frequency of ≥0.90, while there was only one SNV with a frequency of ≥0.90 in the reads that mapped to EBOV Kikwit (Fig. 2B). Thus, we correctly concluded that the precise isolate sequenced in this experiment was EBOV Kikwit.

## DISCUSSION

We describe the successful application of a multiplexed MDS assay and bioinformatics pipelines for comprehensively interrogating the provenance of six virus isolates in a blinded fashion. With regard to multiplexing, the frequency of reads that are assigned an incorrect barcode is a function of the abundance of those reads in the correct barcode library and the degree of multiplexing. So, for high-titer viral stock-derived data sets, where viral reads are abundant, bleed-through occurs at a higher frequency. We demonstrated that dual indexing reduces the misassignment of sequencing reads by 37-fold in a pilot experiment and saw essentially no intersample cross-contamination in our multiplexed assay despite very-high-titer virus isolates. It is important to note that most commercially available dual-indexing kits do not provide this benefit because the reduction in misassignment depends on samples being identified by unique index pairs. Most commercially available kits do not actually provide unique index pairs. Instead, they mix and match indexes to create pairs with repeated index sequences, for instance, by permuting eight i5 indexes with 12 i7 indexes to achieve 96 pairs. We also found that using dual indexing decreased the total number of reads per data set by ~6% due to the removal of unassigned read pairs. However, this is a relatively small tradeoff for the large decrease in misassigned reads.

Contaminating sequences were accurately identified, including bacteria (*Mycoplasma* spp.) and six viruses across the six virus stocks. Four of these viral contaminating sequences (i.e., EBV, HPV18, hamster gammaretrovirus, and LCMV [*n* = 2]) reflected the cells on which the viruses were cultured (10, 11, 13, 14). The fowlpox virus present in the rCDV sample was a remnant of the procedure used to generate rCDV, which uses a recombinant fowlpox virus to express T7 RNA polymerase (5). This is unsurprising given the low passage number of the rCDV.

The results from MDS of different virus stocks highlight the utility of this approach for in-depth analysis of virus sequences. There were different contaminating pathogens in each of the stocks, and these biological “fingerprints” provided precise information about their origin and passage history. Such information is invaluable for virologists embarking on time-consuming and expensive studies in biocontainment but is also useful for a broad range of microbiologists who would benefit from sample assurance. For rMV and rHRSV, there was evidence of the cell lines used to propagate the viruses, and for rCDV, there was evidence of the helper virus used for reverse genetics. For viruses with an extensive propagation history, the cell type in which the virus had been passaged could be inferred based on contaminating sequences (i.e., hamster retrovirus sequences) as well as the additional, nonmicrobial sequences that can be assembled to characterize aspects of the host cell genome. This suggests that MDS is useful for virus forensic analysis, helping to identify the manner and cell type in which the virus was cultured or recovered.

The SNV analysis identified numerous synonymous and nonsynonymous mutations present in each of the isolates. The presence and frequency of such mutations in longitudinal samples may be used to monitor inevitable virus adaptation rigorously in the laboratory setting and the quality of seed stocks (15) as well as to define particular isolates (16). Here, SNV analysis allowed the precise determination of the particular EBOV isolate (Fig. 2B). Last, detailed cataloging of SNVs for each isolate allows rapid identification and tracking of high-priority pathogens in the event of an accidental or nonaccidental release of virus into the environment and could be an integral part of any microbial source-tracking program. Viral genome sequencing including SNV analysis has become an important tool to monitor viral spread, viral evolution, and routes of transmission in an outbreak situation as exemplified by the recent EBOV disease outbreak in West Africa (17–26). If a validated reference sequence does not exist, we recommend that the first step be a *de novo* assembly. However, this should be followed with a remapping of all the reads back to the assembled reference and a LoFreq* or similar SNV analysis, as we have demonstrated here. The latter analysis will reveal the presence of variations or even a mixture of strains that would be obscured or lost through an assemble-and-BLAST strategy alone.

In summary, we show that a single multiplex MDS assay can comprehensively assess (i) virus and isolate identity, (ii) SNVs in virus populations, (iii) the presence of viral coinfection, and (iv) the presence of bacterial contamination and (v) can provide information about the cell line used to propagate the virus.

## MATERIALS AND METHODS

### Viruses and RNA purification.

All work with infectious EBOV was performed under biosafety level 4 (BSL-4) conditions at the Integrated Research Facility, Rocky Mountain Laboratories (RML), Division of Intramural Research, NIAID, NIH, Hamilton, MT. Vero E6 cells (ATCC CRL 1586) were infected with EBOV at a multiplicity of infection (MOI) of 1, and virus-containing supernatants were clarified by low-speed centrifugation at 4 days postinfection (p.i.). Total RNA from supernatant (140 µl) was purified using the Qiagen viral RNA minikit, according to the RML standard operating procedures for virus inactivation. Purified RNA was eluted in H_2_O. The EBOV stock used for this analysis contained a known mycoplasma contamination.

To generate rMV Khartoum, Sudan (rMV^KS^) stocks, B‑LCL cells were infected at an MOI of 0.01. After 4 days, when viral cytopathic effect was maximal, the stock was subjected to one freeze-thaw cycle to release the highly cell-associated measles virus into the medium. Cell debris was removed by low-speed centrifugation, and the supernatant was stored at −80°C as virus stock.

To generate rCDV Rhode Island (rCDV^RI^) stocks, Vero cells expressing the CDV receptor canine CD150 were infected at an MOI of 0.01. After 2 days, when viral cytopathic effect was maximal, the stock was subjected to one freeze-thaw cycle to release cell‑associated virus into the medium. Cell debris was removed by low-speed centrifugation, and the supernatant was stored at −80°C as virus stock.

To generate rHRSV^B05^ stocks, HEp‑2 cells were infected at an MOI of 0.01. After 3 days, when viral cytopathic effect was maximal, the medium was transferred to 50-ml tubes, and cell debris was removed by low-speed centrifugation. The supernatant was stored at −80°C as virus stock.

The LACV H78 strain was grown on BHK cells (27). BHK cells were infected with passage 3 LACV H78 at an MOI of 0.01. Virus was propagated for 48 h before medium was removed. Cell debris was removed by low-speed centrifugation, and individual aliquots of virus were frozen for future analysis. The LACV stock contained a known mycoplasma contamination.

For growth of the VSV Indiana (VSV^IN^) strain, BHK cells were infected with VSV^IN^ at an MOI of 0.01. Virus was propagated for 24 h before medium was removed. Cell debris was removed by low-speed centrifugation, and individual aliquots of virus were frozen for future analysis.

For rMV, rCDV, rHRSV, LACV, and VSV, 250 µl of virus-containing supernatant was mixed with 750 µl of Trizol-LS (Ambion), and RNA was isolated according to the manufacturer’s protocol. Purified RNA was resuspended in H_2_O.

### Comparison of single and dual indexing.

A library containing a pool of 50 dual-indexed libraries was created as previously described (8). The library was sequenced on an Illumina HiSeq 2500 instrument at the UCSF Center for Advanced Technology. The 150-nt read-length, paired-end, dual-indexed data set was manually demultiplexed using either one or both index reads. To measure the rate of read misassignment, reads were first processed to remove low-quality and adapter sequences as described below. Trimmed reads were then aligned using the Bowtie2 tool to the sequence of a reptarenavirus genome segment (GenBank accession no. KP071661.1) actually present in two of the 50 samples (8, 28). Bowtie2 was run with parameters –local –qc-filter –score-min C,120,1. The number of aligning reads for each data set was counted.

### Confirming virus identity and detecting contamination.

RNA of six individual viral isolates extracted from supernatants of infected cells was provided for MDS. The investigators preparing the MDS libraries and performing the bioinformatics analysis were blinded to the presence or absence of any known microbial contaminants and also to the strain and isolate identity of each virus. The six viral isolates were LACV strain H78, EBOV, rCDV^RI^, rMV^KS^, rHRSV^B05^, and the VSV^IN^ strain. Samples were processed for MDS analysis as previously described (9). Samples were randomly amplified to double-stranded cDNA using the NuGEN Ovation v.2 kit (NuGEN, San Carlos, CA), and MDS libraries were constructed using the Nextera protocol (Illumina, San Diego, CA). Each library was dual indexed in the same manner as described above. Samples were pooled into a single library before library size and concentration were determined using the Blue Pippin (Sage Science, Beverly, MA) and Kapa universal quantitative PCR (qPCR) kit (Kapa Biosystems, Woburn, MA), respectively. Samples were sequenced on an Illumina HiSeq 2500 instrument using 150-nt paired-end sequencing. The paired-end sequences were analyzed for microbes using a rapid computational pipeline for microbial detection.

### Bioinformatic pipeline.

Paired-end reads were quality filtered using PriceSeqFilter (version 1.2, parameters –rqf 95 0.98), a component of the paired-read iterative contig extension (PRICE) assembler (29), followed by removal of human sequences by alignment to a combined reference genome including human genome build 38 (hg38) and chimpanzee (*Pan troglodytes*) using the Spliced Transcripts Alignment to a Reference (STAR) aligner (30). Unaligned reads that were at least 95% identical were compressed by cd-hit-dup (v4.6.1) (31, 32). After a second Bowtie2 alignment to remove residual human sequences (using an hg38 reference database), these reads were then used as queries to search the NCBI nt database (July 2015) using gsnapl (Genentech, v2015-09-29) (33).

### SNV analysis.

Illumina data from all the samples were processed in the same way for variant analysis. First reads were quality filtered using PriceSeqFilter (version 1.2) set to remove any read with less than 95% of nucleotides having a 0.98 probability of being correct (-rqf 95 0.98), any read with less than 90% called nucleotides, and any read that matched to the Illumina adapter sequences (29). Filtered high-quality reads were then aligned to reference genomes using GSNAP (version 2015-09-29) using default settings (33). Reference genome accession numbers were as follows: EBOV, JQ352763; LACV long (L) segment, NC_004108; LACV medium (M) segment, NC_004109; LACV short (S) segment, NC_004110; MV, HM439386; rHRSV, KF640637; VSV^IN^, J02428. The rCDV^RI^ sequence (unpublished) was supplied by W. Paul Duprex (Boston University). According to the recommended guidelines for the variant caller, PCR-duplicate reads were removed using Picard tools (version 2.2.4). Variants were called using LoFreq* (version 2.1.2) (34) using default settings with the exception of a conservative lower cutoff of ≥0.005 in frequency. LoFreq* is a sequencing read quality aware variant caller that models each location in the genome as a Poisson-binomial distribution of the number of nonreference bases. It also tests for common sequencing errors that may result in false positives, such as testing for the strand bias of a variant. It is also designed to take advantage of high-coverage (>500× coverage) data sets, such as those that we have (Table 2). It uses high-coverage data sets to call true-positive low-frequency variants while maintaining a low false-positive rate but still shows reasonable sensitivity at low coverage levels (50× coverage) (34). Variants were determined to be synonymous or nonsynonymous using a custom Python script.

### Accession number(s).

The 150-nt paired-end sequences are located in the National Center for Biotechnology Information (NCBI) Short Read Archive (SRA accession number SRP076690).
